# Acceleration of spontaneous visual recovery by voluntary physical exercise in adolescent amblyopic rats

**DOI:** 10.3389/fncel.2024.1519197

**Published:** 2024-12-20

**Authors:** Irene Di Marco, Gabriele Sansevero, Nicoletta Berardi, Alessandro Sale

**Affiliations:** ^1^Neuroscience Institute National Research Council (CNR), Pisa, Italy; ^2^NEUROFARBA, University of Florence, Florence, Italy

**Keywords:** amblyopia, physical activity, visual cortex plasticity, adolescent rats, monocular deprivation

## Abstract

Abnormal visual experience during development resulting from an imbalance in the activity of the two eyes can lead to permanent severe visual deficits, a pathology called amblyopia (lazy eye). While this condition is extremely difficult to treat in adults, current interventions can elicit significant amounts of visual recovery when performed in juveniles before the end of the critical period, even if the achievable results can be unsatisfactory due to the progressive decline in visual cortical plasticity. Similarly to human subjects, rodents becoming amblyopic due to early visual deprivation can display spontaneous functional recovery if the deprivation ends within the critical period time window. With the aim to investigate the impact of non-invasive strategies able to increase this spontaneous potential for plasticity, we wondered whether physical exercise could speed up spontaneous recovery of visual functions in juvenile amblyopic rats. Our results show that physical exercise accelerates visual recovery in adolescent rats, encouraging application of behavioral plasticizing treatments to promote recovery in young individuals.

## 1 Introduction

Amblyopia (lazy eye) is a neurodevelopmental disorder arising from abnormal visual experience early in life, mainly in one eye, due to strabismus, anisometropia, or deprivation (Holmes and Clarke, 2006a). Amblyopia results in permanent changes in the primary visual cortex (V1), where neuronal connections undertake a pronounced reshaping in favor of the healthy eye (Antonini et al., 1999; Holmes and Clarke, 2006b; Sengpiel, 2014; Wiesel and Hubel, 1963), eventually leading, at the functional level, to low visual acuity in the amblyopic eye and severe deficits in depth perception abilities (Holmes and Clarke, 2006a). It is widely accepted that amblyopia can be corrected only whether the intervention, primarily based on the occlusion/penalization of the healthy eye to force the use of the amblyopic one, is performed before the closure of the critical period (CP) for ocular dominance plasticity and visual acuity development (Fronius et al., 2014; Kehrein et al., 2016; Mehmed et al., 2022). Treatments performed in adolescents and adults, indeed, do not result in any significant improvement (Brin et al., 2021; Papageorgiou et al., 2019; Zhou et al., 2006).

In recent years, several behavioral and pharmacological interventions have been proved effective in eliciting visual cortex plasticity and visual function recovery past the closure of the CP, both in animal models and in human patients (Duffy et al., 2023; Papageorgiou et al., 2019; Sale and Berardi, 2015; Stryker and Löwel, 2018). Among the emerging treatments, one condition that appears particularly promising for suitable clinical applications is physical exercise, a strong regulator of brain plasticity (Augusto-Oliveira et al., 2023; Consorti et al., 2021). We previously showed that 3 weeks of voluntary physical exercise induced a marked recovery of visual acuity and depth perception abilities in adult amblyopic rats, either when the animals were maintained in reverse-suture conditions (i.e., closing of the healthy eye, while the amblyopic eye is open) (Baroncelli et al., 2012), or after reestablishing binocular sight conditions (Sansevero et al., 2020). Moreover, cycling in a stationary bicycle elicited robust visual plasticity in adult healthy human subjects as well (Lunghi and Sale, 2015).

While application of physical exercise interventions to adult amblyopic subjects has been successfully exploited (see Lunghi et al., 2019), to date the possibility to adapt this procedure for boosting plasticity during the CP has never been investigated. A similar approach has a strong rationale, as it has been shown that, in rodents, brief periods of eye deprivation during the CP, a procedure leading to amblyopia, can be reversed if the eyelids are reopened and normal binocular sight conditions are reestablished before the end of the CP (Hofer et al., 2006). Thus, a procedure capable of enhancing the spontaneous potential for plasticity might have a beneficial effect on amblyopia recovery. Here, we investigated for the first time the impact of physical exercise on the time course of visual function rescue in adolescent rats that were rendered amblyopic by a brief period (7 days) of monocular deprivation (MD) performed at the peak of the CP.

## 2 Methods

### 2.1 Animal treatment and surgical procedures

The experiments were conducted on Long-Evans black hooded rats. All experimental protocols were approved by the Italian Ministry of Public Health (approved protocol n. 23C). The animals were housed in a room with a temperature of 21°C and a 12-h light–dark cycle, with food and water available ad libitum.

### 2.2 Surgical and experimental procedures

Rats were anesthetized with zolazepam + tiletamine (Zoletil, 1 mg/kg) and monocular deprivation (MD) was performed through eyelid suture at postnatal day (P) 21. Lid margins were trimmed and sutured with 6–0 silk. A post-operative health check control was performed daily until complete recovery.

For the first experiment 12 rats were rendered amblyopic by MD for 7 days (from P21 to P28). At P28, the deprived eye was reopened under anesthesia using thin scissors, and rats were divided into two groups: Runners (RUN, *n* = 6) and Sedentary (SED, *n* = 6). A third group of naïve, age-matched animals (NAÏVE, *n* = 5) was added as control. These animals were used to evaluate visual functions recovery at the age of P43.

**Figure 1:**
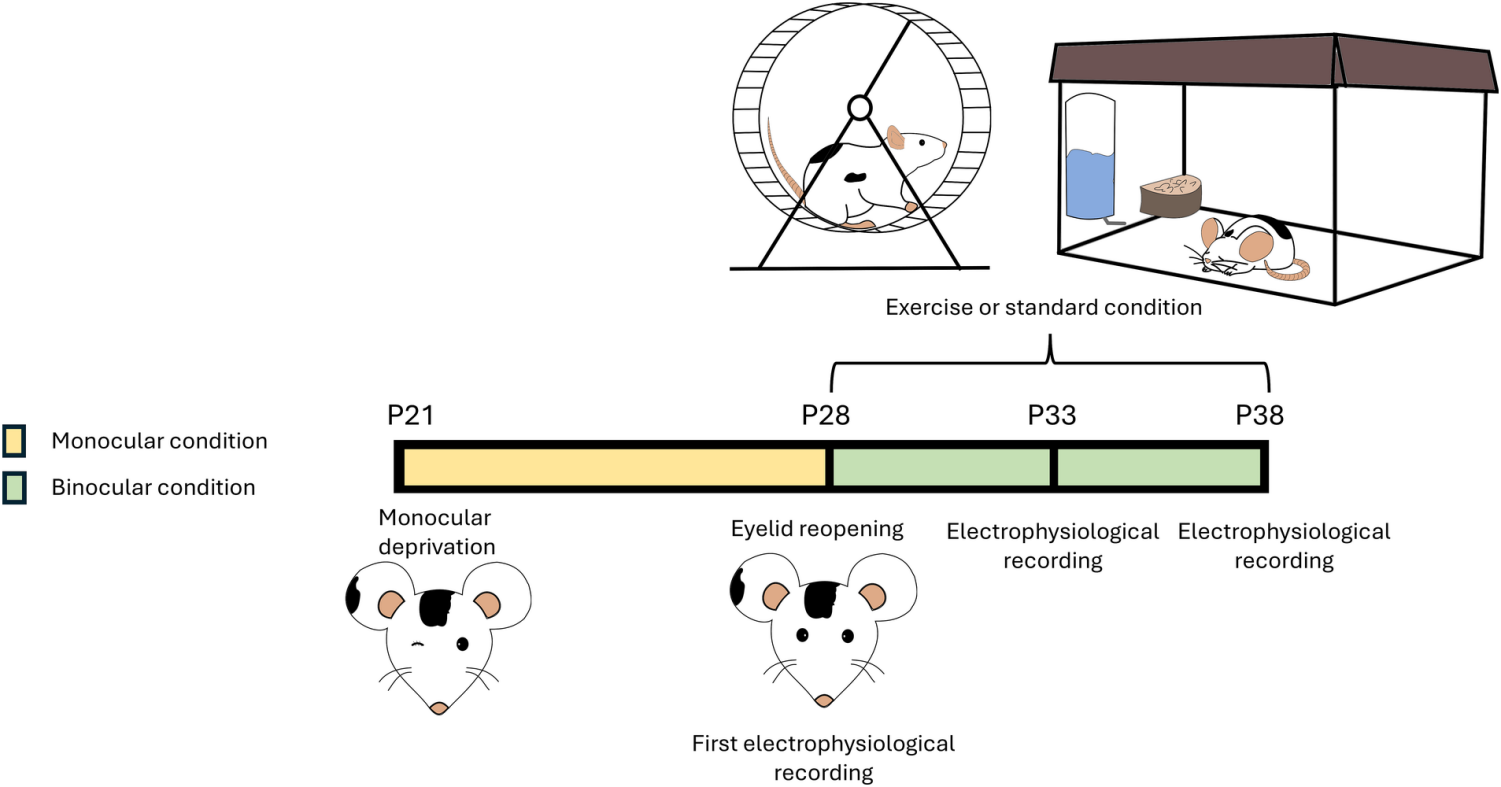
Schematic representation of the protocol. A group of rats were rendered amblyopic through 7 days monocular deprivation (MD) (from postnatal day (P) 21 to P28). At P28, the deprived eye was reopened. After reopening of the deprived eye, rats were divided into two groups: the running group had free access to a wheel and could perform voluntary physical exercise (Runner), while the sedentary group was reared in conventional standard conditions (Sedentary). Both groups were maintained in binocular sight conditions. Rats underwent electrophysiological recordings every 5 days (at P28, P33, and P38). A group of non-deprived naïve rats, subjected to the same electrophysiological recordings (at P28, P33, and P38), served as control.

For the second experiment, a group of 12 different rats were used to longitudinally assess visual function recovery at three ages, P28, P33, P38. At P28, the deprived eye was reopened under anesthesia using thin scissors, and a cranial window was created in the binocular zone of the primary visual cortex (V1, 4.8–5.2 mm lateral to lambda), contralateral to the deprived eye. On the same day, electrophysiological recordings were conducted (pre-treatment, at P28). After the recording, the cranial window was filled with agar to keep the cortex moist, then sealed with Kwik-Cast silicone elastomer (World Precision Instruments, USA) to isolate the cerebral tissue from the external environment. Dental cement (Paladur^®^) was applied to the exposed cranial bone. After reopening of the deprived eye, the rats were divided into two groups: the running group (*n* = 6), which had free access to a wheel for voluntary physical exercise (RUN), and the sedentary group (*n* = 6), which was housed under standard conditions (SED) (Figure 1). A third group of naïve (NAÏVE), age-matched animals (*n* = 5) was added as controls.

SED and NAÏVE animals were individually maintained in standard housing conditions, consisting of 40 × 25 × 20 cm cages. RUN rats were individually placed in cages endowed with a running wheel.

### 2.3 *In vivo* electrophysiology

Rats were anesthetized by an intraperitoneal injection of a mixture of Zolazepam and Tiletamine (Zoletil-100, 40 mg/kg, Virbac) and Xylazine (Xilor, 10 mg/kg, Sigma) and placed in a stereotaxic frame, with the body temperature maintained at 37°C. An electrode (A1x16-3 mm-50-703-A16, Neuronexus Technologies) was lowered into the cortex to record local field potentials. Signals were acquired using a 16-channel Open Ephys device, and data analysis was performed using a custom made Python software. Visual stimuli were generated in Python with the PsychoPy extension and displayed on a monitor (Asus VG278QR, 165 Hz refresh rate, 400 cd m^−2^ mean luminance) placed 20 cm in front of the animal.

For visual evoked potentials (VEPs), the extracellular signal was filtered from 0.3 to 275 Hz and sampled at 30.3 kHz. VEPs in response to sinusoidal wave gratings with a spatial frequency of 0.06 c/deg and abrupt phase inversion (2 Hz temporal period) were evaluated in the time domain by measuring the peak-to-baseline amplitude and latency. The Contralateral/Ipsilateral (C/I) VEP ratio, a measure of the ocular dominance (OD), was obtained calculating the ratio of VEP amplitudes recorded during the presentation of low frequency gratings (0.06 c/deg) to the eye contralateral and ipsilateral, respectively, to the visual cortex where the recording was performed. In normal rats, the C/I VEP ratio is between 2.0 and 3.0, but decreases to around 1.0 after MD, reflecting the OD shift in favor to the non-deprived eye (Baroncelli et al., 2012; Sale et al., 2007). For visual acuity, sinusoidal wave gratings were used as visual stimuli (30 repetitions of the stimulus, temporal frequency of 2 Hz, 7 different spatial frequencies (0.1, 0.2, 0.3, 0.4, 0.5, 0.6, 0.7 c/deg), contrast and luminance maintained fixed at 100%). Visual acuity was evaluated by extrapolation to zero amplitude of the linear regression through the data points in a curve where VEP amplitude is plotted against log spatial frequency.

Electrophysiological recordings were conducted at P43 in the first experiment (*n* = 17) and every 5 days (at P28, P33, and P38) in the second experiment (*n* = 17).

### 2.4 Statistical analysis

R (version 4.1.2) was used for statistical analysis. Data were tested for normality before running the statistical tests. Parametric tests were conducted on normally distributed data; if the normality test failed, non-parametric tests were performed as appropriate. Differences between two independent groups were assessed with a two-tailed t-test; differences between two dependent groups were assessed with a two-tailed paired t-test. One-way ANOVA, One-way RM ANOVA, and Two-way RM ANOVA were used to compare normally distributed data from multiple groups. One-way ANOVA on ranks or Two-way ANOVA on ranks was performed to compare non-normally distributed data from more than two groups. In the results section, all data are expressed as the mean ± standard error of the mean (S.E.M.). Correlation was computed using the Pearson’s correlation coefficient (r); statistical significance was assessed using the two-tailed t-test.

## 3 Results

We first investigated the degree of spontaneous recovery from the effects of MD after 15 days of eye reopening in sedentary animals. Rats (*n* = 12) rendered amblyopic through 7 days of MD (from P21 to P28) were subjected to eye reopening at P28 and then left to recover under sedentary conditions (SED, *n* = 6) or under running conditions, for comparison (RUN, *n* = 6). Naïve, age-matched animals (NAÏVE, *n* = 5) were added as controls. Visual recovery was assessed by means of electrophysiological recordings from V1, evaluating visual acuity and ocular dominance using visual evoked potentials (VEPs) at P43.

**Figure 2:**
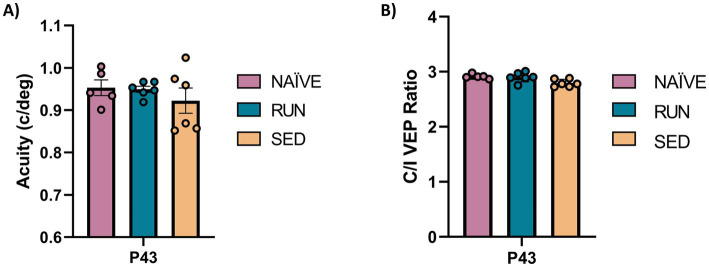
Spontaneous visual function recovery from amblyopia in adolescent rats. **(A)** Electrophysiological recordings of visual evoked potentials from the primary visual cortex. At P43 there was no difference in the visual acuity (c/deg) in all tested groups (One-way ANOVA, *p* = 0.582). **(B)** Ocular dominance was assessed through the Contralateral/Ipsilateral (C/I) VEP ratio in response to a low spatial frequency grating. At P43 there was no difference in C/I VEP ratio in all tested group (One-way ANOVA, *p* = 0.999).

At P43, after 2 weeks of binocular experience, visual recovery was complete both in adolescent SED and RUN animals, with their visual acuity not different from that of NAÏVE animals (SED deprived eye visual acuity vs. NAÏVE contralateral eye, 0.922 ± 0.029 vs. 0.953 ± 0.018: One-way ANOVA, Tukey method, DF = 2, *F* = 0.638, *p* = 0.582; SED deprived eye at P43 vs. RUN deprived eye at P43, 0.922 ± 0.029 vs. 0.949 ± 0.007: One-way ANOVA, *p* = 0.633, Figure 2A). At this time point, SED rats also displayed a complete recovery of the C/I VEP ratio, with no difference with respect to either NAÏVE (One-way ANOVA, Tukey method, DF = 2, *F* = 5.103; NAÏVE ratio at P43 vs. SED ratio at P43, 2.913 ± 0.018 vs. 2.794 ± 0.027, *p* = 0.999) or RUN animals (One-way ANOVA, RUN ratio at P43 vs. SED ratio at P43, 2.902 ± 0.036 vs. 2.794 ± 0.027, *p* = 0.999, Figure 2B).

Then, to investigate the capability of voluntary physical activity to accelerate visual function recovery in adolescent amblyopic rats, we performed an additional experiment with different rats in order to longitudinally follow visual improvements in both SED (*n* = 6) and RUN (*n* = 6) rats. A group of NAÏVE animals was also included as further control. The first electrophysiological recordings occurred at P28, immediately after eye reopening (pre-treatment), and then we performed follow-up assessments every 5 days (at P33 and P38; Figure 1).

**Figure 3:**
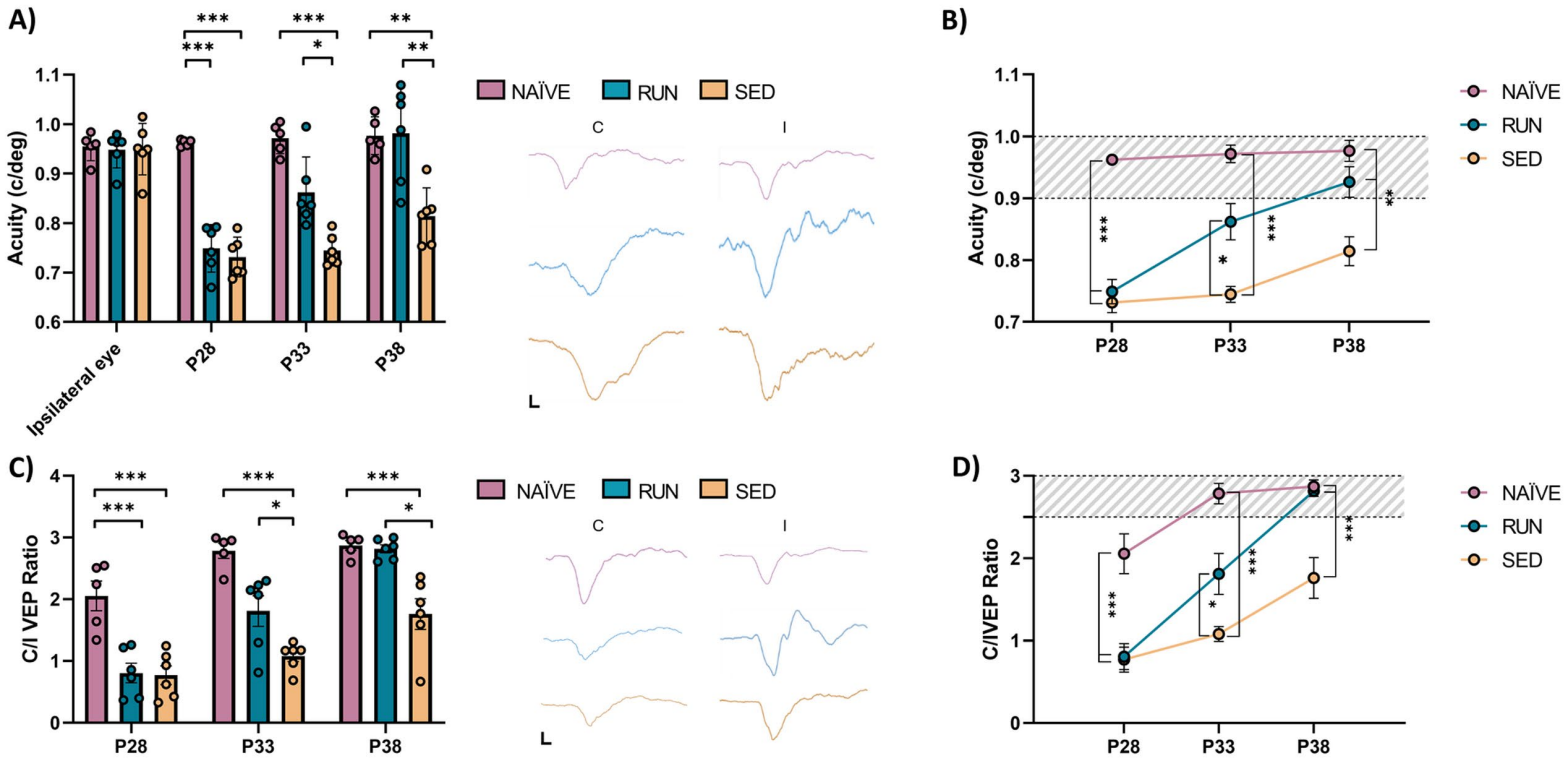
Longitudinal visual assessment. **(A,B)** Electrophysiological recordings of visual evoked potentials from the primary visual cortex. There was no difference in the visual acuity (c/deg) of the fellow eye at P28 (Two-way RM ANOVA, Tukey method, *p* = 1.00). Ten days of free running led to a full recovery in the amblyopic eye in the Runner group, that showed values not different from those of naïve animals (Two-way RM ANOVA, *p* = 1.00). In contrast, Sedentary rats showed values significantly lower with respect to those of naïve animals (Two-way RM ANOVA, *p* < 0.001). VEP traces between the two graphs were obtained by presenting gratings with a spatial frequency of 0.1 c/deg to the contralateral and ipsilateral eye at P28. The black bars indicate the scale (*x*-axis = 50 ms; *y*-axis = 100 μV). **(C,D)** Ocular dominance was assessed through the Contralateral/Ipsilateral (C/I) VEP ratio in response to a low spatial frequency grating. At P38, the C/I VEP ratio in the Runner group did not differ from that of naïve animals (One-way ANOVA, Tukey method, *p* = 1.00). VEP traces between the two graphs were obtained by presenting gratings with a spatial frequency of 0.06 c/deg to the contralateral and ipsilateral eye at P28. The black bars indicate the scale (*x*-axis = 50 ms; *y*-axis = 100 μV). (P28, P33, P38, P43 = Postnatal day 28, 33, 38, 43; *p < 0.05; **p < 0.01; **p < 0.001; error bars indicate S.E.M.).

At P28, we found that MD led to a reduction in visual acuity in the deprived eye compared to the fellow eye in all animals (prospective SED rats, *n* = 6, Two-way ANOVA RM, Tukey method, eye × group, DF = 6, *F* = 14.78, fellow eye vs. deprived eye at P28, 0.949 ± 0.021 vs. 0.731 ± 0.016, *p* < 0.001; prospective RUN rats, *n* = 6, Two-way ANOVA RM, 0.948 ± 0.015 vs. 0.749 ± 0.019, *p* < 0.001; Figures 3A,B). In NAÏVE rats, there was no difference in visual acuity between the two eyes at the same age (*n* = 5, Two-way ANOVA RM, contralateral vs. ipsilateral, 0.954 ± 0.013 vs. 0.962 ± 0.003, *p* = 0.999). The first 5 days of free running resulted in a significant increase in visual acuity in the RUN group (deprived eye at P28 vs. deprived eye at P33, 0.749 ± 0.019 vs. 0.862 ± 0.029, Two-way ANOVA RM, *p* = 0.0016); 5 days of additional running led to complete recovery of visual acuity in the previously deprived eye, with no significant difference compared to the fellow eye (fellow eye vs. deprived eye at P38, 0.948 ± 0.015 vs. 0.981 ± 0.039, Two-way ANOVA RM, *p* = 0.963) or to the visual acuity of NAÏVE rats (RUN deprived eye at P38 vs. NAÏVE contralateral eye at P38, 0.981 ± 0.039 vs. 0.976 ± 0.017, Two-way ANOVA RM, *p* = 1.000, Figures 3A,B). On the contrary, in SED rats (*n* = 6) 15 days of binocular vision led to a progressive increase in visual acuity (deprived eye at P28 = 0.731 ± 0.016 c/deg vs. deprived eye at P38 = 0.814 ± 0.023 c/deg, Two-way ANOVA RM, *p* = 0.053) which, however, remained significantly lower compared to that of both NAÏVE and RUN rats (SED deprived eye at P38 vs. NAÏVE contralateral eye at P38, 0.814 ± 0.023 vs. 0.976 ± 0.017, Two-way ANOVA RM, *p* = 0.0003; SED deprived eye at P38 vs. RUN deprived eye at P38, 0.814 ± 0.023 vs. 0.981 ± 0.039, Two-way ANOVA RM, *p* = 0.0001, Figures 3A,B).

**Figure 4:**
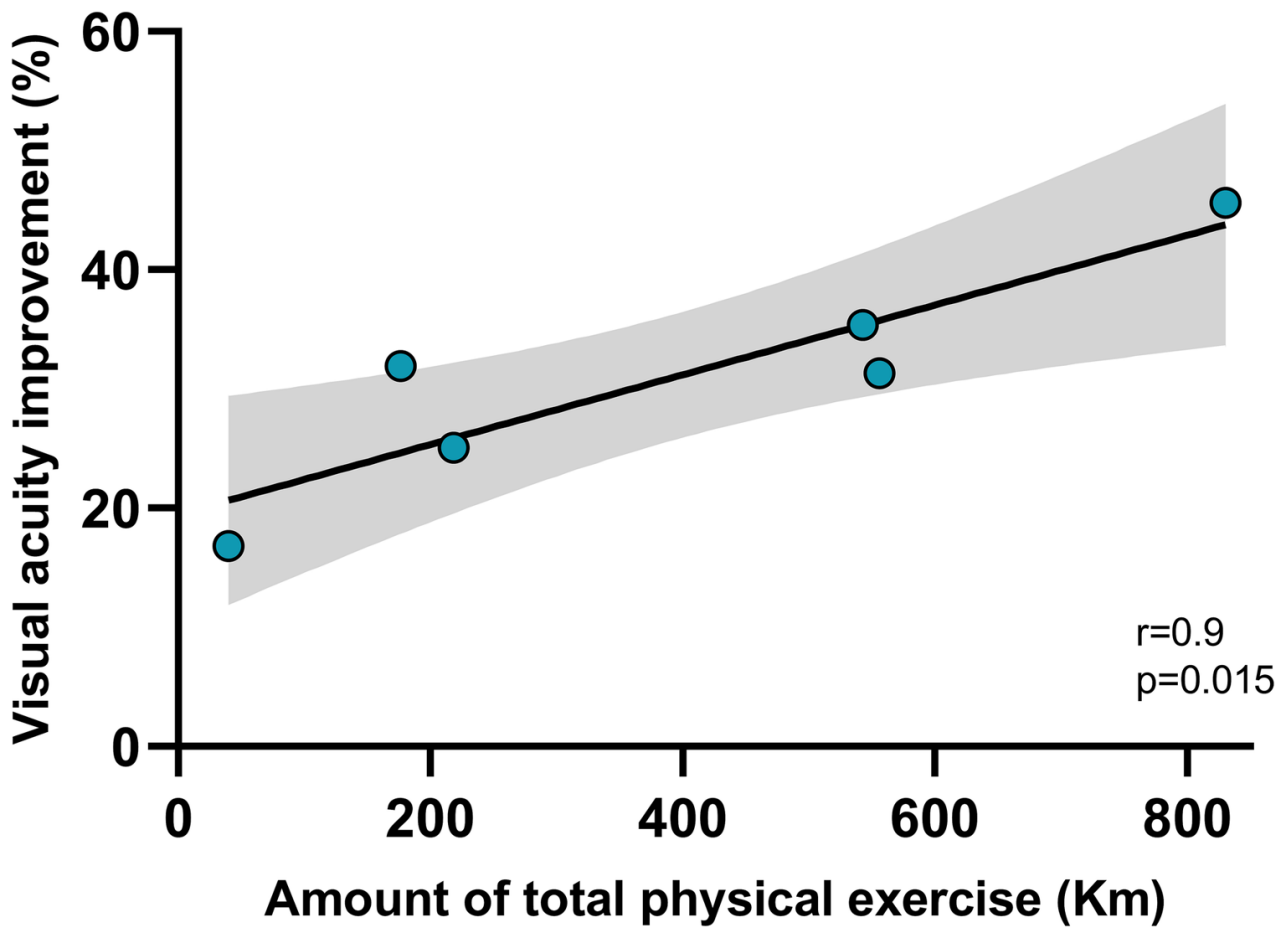
Correlation between the amount of physical exercise and visual acuity improvement. The amount of physical exercise was measured as the average distance (in Km) run by each rat between P28 and P38 (*x*-axis). The visual acuity improvement was calculated as the percentage change between the acuity value measured at the reopening of the eye after one week of monocular deprivation (P28), and the value measured after 15 days of voluntary physical exercise (P38). The correlation resulted significant (*p* < 0.05). The gray zone represents the confidence interval.

Thus, running determined an acceleration of visual recovery, with typical visual acuity recovered after 10 days. Importantly, we detected a strong positive correlation between the individual amount of visual acuity improvement and that of physical activity performed by the animals. Indeed, as the amount of voluntary physical exercise increased, there was a more marked improvement in the degree of visual acuity recovery (two tailed t-test, DF = 4, *t* = 4.061, *p* < 0.05; Figure 4).

Additionally, we observed a significant increase of the contralateral/ipsilateral (C/I) VEP ratio in RUN rats at P33 (*n* = 6, P28 = 0.806 ± 0.157 vs. P33 = 1.810 ± 0.248, Two-way ANOVA RM, time × group, DF = 4, *F* = 6.353, *p* < 0.001) with a complete recovery at P38 (P28 = 0.806 ± 0.157 vs. P38 = 2.816 ± 0.066, Two-way ANOVA RM, *p* < 0.001), with no difference compared to NAÏVE rats (Two-way ANOVA RM, NAÏVE ratio at P38 vs. RUN ratio at P38, 2.872 ± 0.079 vs. 2.816 ± 0.066, *p* = 1.000, Figures 3C,D). SED animals at P33 did not achieve any increase in C/I VEP ratio (P28 = 0.770 ± 0.150 vs. P33 = 1.081 ± 0.090, Two-way ANOVA RM, *p* = 0.783). 10 days after eyelid reopening, an increase in C/I VEP ratio was observed (P28 = 0.770 ± 0.150 vs. P38 = 1.761 ± 0.248, Two-way ANOVA RM, *p* = 0.0006); however, this value did not reach that of NAÏVE rats (Two-way ANOVA RM, NAÏVE ratio at P38 vs. SED ratio at P38, 2.872 ± 0.079 vs. 1.761 ± 0.248, *p* = 0.0024, Figures 3C,D).

**Figure 5:**
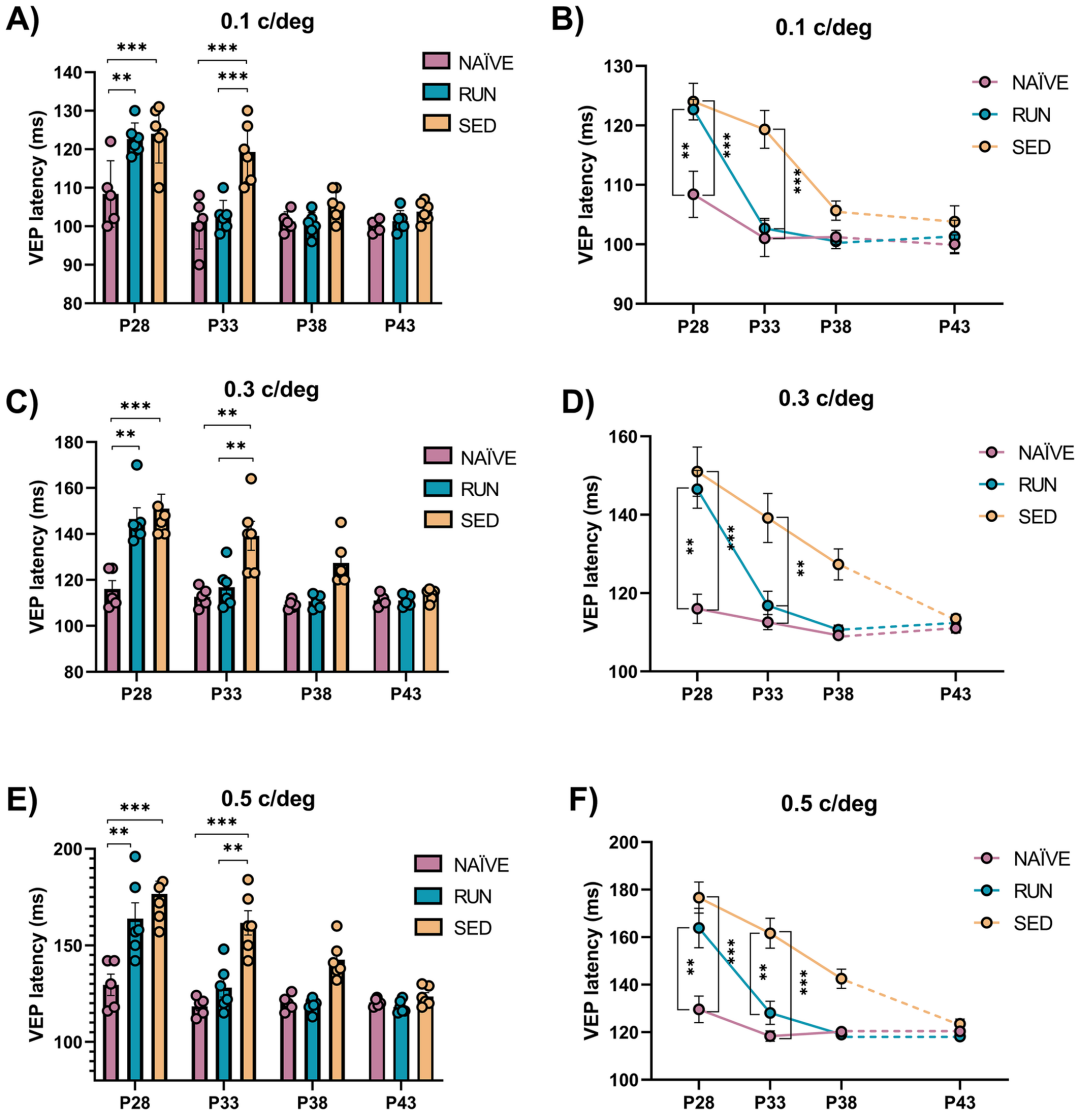
Electrophysiological P/N1 latency assessment. **(A,B)** 0.1 c/deg At P28 VEP latency in response to sinusoidal wave gratings at 0.1 c/deg of perspective Runner and perspective Sedentary rats was increased with respect to Naïve animals (Two-way RM ANOVA, Tukey method, *p* < 0.001 for all comparison). At P33 VEP latency in Runner rats did not differ from that of Naïve rats (Two-way ANOVA RM, *p* = 0.999), while Sedentary rats had VEP latencies significantly higher than that of Naïve (Two-way ANOVA RM, *p* < 0.001) and Runner (Two-way ANOVA RM, *p* < 0.001) rats. VEP latencies at P38 and P43 were not different among Naïve, Runner and Sedentary animals (Two-way ANOVA RM, *p* > 0.1). **(C,D)** 0.3 c/deg At P28, VEP latency in response to sinusoidal wave gratings at 0.3 c/deg was increased in both perspective Sedentary and perspective Runner rats compared to Naïve animals (Two-way RM ANOVA, Tukey method, *p* < 0.001 for Sedentary and *p* < 0.01 for Runner). At P33, VEP latency Runner decreased and reached that of Naïve rats (Two-way ANOVA RM, *p* = 0.999), while VEP latency in Sedentary rats was still significantly higher than that of Naïve (Two-way ANOVA RM, *p* < 0.01) and Runner (Two-way ANOVA RM, *p* < 0.05) rats. VEP latency since P38 onward was not different among Naïve, Runner and Sedentary animals (Two-way ANOVA RM, *p* > 0.1). **(E,F)** 0.5 c/deg At P28, the VEP latency in response to sinusoidal wave gratings at 0.5 c/deg was increased in both perspective Sedentary and perspective Runner rats compared to Naïve animals (Two-way RM ANOVA, Tukey method, *p* < 0.01 for Sedentary and *p* < 0.01 for Runner). At P33 there was no difference in the VEP latency between Runner and Naïve rats (Two-way ANOVA RM, *p* = 0.963), while VEP latency of Sedentary rats was still significantly higher than that of Naïve (Two-way ANOVA RM, *p* < 0.001) and Runner (Two-way ANOVA RM, *p* < 0.001) rats. VEP latency at P38 and P43 were not different among Naïve, Runner and Sedentary animals (Two-way ANOVA RM, *p* > 0.1). (P28, P33, P38, P43 = Postnatal day 28, 33, 38, 43; *p < 0.05; **p < 0.01; **p < 0.001; error bars indicate S.E.M.).

The latency of the first VEP component (P/N1) is a robust indicator of early visual cortex development (Narducci et al., 2018). Thus, we measured, at the same ages used to monitor visual acuity, VEP latency for spatial frequencies of 0.1, 0.3, and 0.5 c/deg. We found a marked impairment in the maturation of VEP latencies in perspective SED rats compared to NAÏVE animals, with higher VEP latencies observed for all the spatial frequencies tested at P28 (Two-way ANOVA RM, Tukey method, time × group, DF = 4, *F* = 7.052, 0.1 c/deg at P28 NAÏVE vs. SED, 106.4 ± 4.166 vs. 124 ± 3.087, *p* < 0.001, Figures 5A,B; 0.3 c/deg at P28 NAÏVE vs. SED, 118 ± 4.939 vs. 151 ± 6.292, *p* < 0.01 Figures 5C,D; 0.5 c/deg at P28 NAÏVE vs. SED, 133.6 ± 8.495 vs. 176.67 ± 6.556, *p* < 0.01, Figures 5E,F). Also the group of perspective RUN rats at P28 showed an impairment in the maturation of VEP latencies compered to NAÏVE animals (Two-way ANOVA RM, 0.1 c/deg at P28 NAÏVE vs. RUN, 106.4 ± 4.166 vs. 122.67 ± 1.706, *p* < 0.01, Figures 5A,B; 0.3 c/deg at P28 NAÏVE vs. RUN, 118 ± 4.939 vs. 136.67 ± 7.007, *p* < 0.01 Figures 5C,D; 0.5 c/deg at P28 NAÏVE vs. RUN, 133.6 ± 8.495 vs. 158.83 ± 10.074, *p* < 0.01, Figures 5E,F). However, RUN rats achieved a complete recovery of VEP latencies at P33, with values shorter than those of SED rats (Two-way ANOVA RM, 0.1 c/deg at P33 RUN vs. SED, 102.67 ± 1.667 vs. 129.33 ± 3.169, *p* < 0.001, Figures 5A,B; 0.3 c/deg at P33 RUN vs. SED, 116.83 ± 3.618 vs. 140.67 ± 5.63, *p* < 0.05, Figures 5C,D; 0.5 c/deg at P33 RUN vs. SED, 128.17 ± 4.895 vs. 161.67 ± 6.269, *p* < 0.001; Figures 5E,F), and equal to those of NAÏVE rats (Two-way ANOVA RM, 0.1 c/deg at P33 NAÏVE vs. RUN, 101 ± 3.066 vs. 102.67 ± 1.667, *p* = 0.999, Figures 5A,B; 0.3 c/deg at P33 NAÏVE vs. RUN, 112.6 ± 1.913 vs. 116.83 ± 3.618, *p* = 0.999, Figures 5C,D; 0.5 c/deg at P33 NAÏVE vs. RUN, 118.4 ± 2.159 vs. 128.17 ± 4.895, *p* = 0.963; Figures 5E,F). In SED rats, this impairment persisted at P33 (Two-way ANOVA RM, Tukey, method, time × group, DF = 4, *F* = 3.139, 0.1 c/deg at P33 NAÏVE vs. SED, 101 ± 3.066 vs. 129.33 ± 3.169, *p* < 0.001, Figures 5A,B; 0.3 c/deg at P33 NAÏVE vs. SED, 112.6 ± 1.913 vs. 140.67 ± 5.63, *p* < 0.01, Figures 5C,D; 0.5 c/deg at P33 NAÏVE vs. SED, 118.4 ± 2.159 vs. 161.67 ± 6.269, *p* < 0.001, Figures 5E,F). At P38, VEP latencies of SED rats became equal to those of NAÏVE animals (Two-way ANOVA RM, Tukey method, time × group, DF = 4, *F* = 3.015, 0.1 c/deg at P38 NAÏVE vs. SED, 101.2 ± 1.157 vs. 105.67 ± 1.605, *p* = 0.937, Figures 5A,B; 0.3 c/deg at P38 NAÏVE vs. SED, 109.2 ± 0.860 vs. 127.33 ± 3.964, *p* = 0.192, Figures 5C,D; 0.5 c/deg at P38 NAÏVE vs. SED, 120.2 ± 1.855 vs. 142.5 ± 4.039, *p* = 0.224; Figures 5E,F). Thus, these results show that MD affects maturation of VEP latencies, but its effects can be effectively counteracted by physical exercise.

## 4 Discussion

While there is increasing consensus on the need to develop suitable strategies to treat amblyopia in adult subjects (Yeritsyan et al., 2024), and great effort is devoted to this goal, in current clinical practice the gold-standard approach remains in children, that are treated by monocularly patching or other forms of penalizing the non-amblyopic eye (Holmes and Clarke, 2006b). Early treatment in children can lead to robust visual acuity rescue, but many patients do not respond adequately. Moreover, recent evidence is leading to an increasing consensus on the relevance of binocular therapeutic approaches to facilitate visual processing from the amblyopic eye (Thompson et al., 2024).

In this study, we focus on the possibility to use a widely employed plasticizing treatment, based on an increase in voluntary physical exercise levels, to accelerate visual function recovery in young animals with restored binocular sight conditions around the end of their CP for visual cortex plasticity. We show, in a group of rats with free access to a running wheel for voluntary physical exercise, that 10 days of this regimen were sufficient to achieve complete recovery of visual acuity and to restore normal values in the C/I VEP ratio. Notably, we found a dose–response effect, as there was a strong positive correlation between acuity improvement and the amount of voluntary physical exercise. This result, even if limited by the small sample size of the group tested, suggests a direct causal influence of physical exercise on visual recovery.

The latency of VEPs is a very reliable index of visual system development, both in animal models and in humans (Guzzetta et al., 2009; Lee et al., 2012). Moreover, it has been shown that VEP latency maturation is strongly sensitive to the environmental conditions, with slower decreases in latency of VEP responses and slower myelination rates in developing rats exposed to impoverished conditions (Narducci et al., 2018). Here, we found that one week of MD resulted in increased P/N1 VEP latencies in adolescent rats compared to naïve animals. This effect was consistent across all tested spatial frequencies (0.1, 0.3, and 0.5). Remarkably, rats that performed voluntary physical exercise achieved VEP latencies comparable to naïve animals within 5 days of the treatment. In contrast, sedentary rats took twice as long to achieve the same amount of recovery. Interestingly, restoration of VEP latencies was evident before the improvement in visual acuity or C/I VEP ratio values, indicating that different functional properties of the visual system may recover at different rates.

Despite the growing interest in the benefits of physical exercise for brain plasticity, the mechanisms underlying its impact on visual function recovery are still unclear. Increasing evidence suggests that motor activity may enhance the response of visual cortical neurons to visual stimuli by activating specific GABAergic neural circuits that increase visual gain in response to visual stimulation (Kaneko and Stryker, 2014; Sale et al., 2010; Sansevero et al., 2020). It is interesting to notice that the animals were allowed to exert voluntary physical exercise both during the day and in the night. During the light phase of the day, the natural visual stimulation from the surroundings added to the strong visual stimulation arising from the running wheel. While this was also possible during the dark phase of the day (as darkness was not complete), it is likely that the amount of visual stimulation was reduced during the night. In both conditions, running has been previously shown to exert a strong impact on GABAergic neural circuits (see Sansevero et al., 2020).

In parallel to this effect at the level of the central nervous system, the beneficial impact of physical exercise on V1 plasticity may also be associated with a peripheral stimulation of mild levels of oxidative stress and circulating factors such as IGF-1, eventually enhancing brain plasticity (Sansevero et al., 2023). Further studies will need to focus on the mechanisms underlying the capability of physical exercise to promote visual functional recovery in adolescent subjects.

Our findings were obtained in rats rendered amblyopic by MD and then subjected to restoration of normal binocular conditions. Thus, they can have significant implications for the development of suitable treatment strategies for amblyopic subjects that do not involve the occlusion of the healthy eye. Indeed, occluding one eye could hinder restoration of depth perception, and wearing an eye patch in juveniles is purely tolerated, due to its remarkable impact for social relationships.

Interestingly, short-term deprivation (i.e., 150 min) has been shown to enhance the response of the deprived eye in human subjects, as tested with a binocular rivalry perception task (Lunghi et al., 2011). This strategy has been exploited to treat amblyopia in a new, counterintuitive, manner: occluding the amblyopic eye while intermittently cycling on a stationary bike promotes recovery of both visual acuity and depth perception abilities in adult amblyopic subjects (Lunghi et al., 2019). Based on the results shown in the present study, future research should investigate the impact of such a combined approach of physical exercise + short occlusion of the amblyopic eye in children and adolescent patients.

In conclusion, our study emphasizes the need to develop new non-invasive interventions to counteract amblyopia, a severe condition with still a remarkable negative impact for the patients’ life.

## Data Availability

The raw data supporting the conclusions of this article will be made available by the authors, without undue reservation.
